# Massive infected pancreatic necrosis in an 8‐year‐old: Endoscopic management

**DOI:** 10.1002/jpr3.12052

**Published:** 2024-02-14

**Authors:** William F. Abel, Varun Kesar, Reid D. Wasserman, Manoj Kumar, Vishal Patel, Paul Yeaton, Vivek Kesar

**Affiliations:** ^1^ Virginia Tech Carilion, Department of Internal Medicine Roanoke Virginia USA; ^2^ Virginia Tech Carilion, Department of Internal Medicine, Division of Gastroenterology Roanoke Virginia USA; ^3^ Tufts Medical Center, Department of Internal Medicine, Division of Gastroenterology Boston Massachusetts USA; ^4^ Virginia Tech Carilion, Department of Radiology Roanoke Virginia USA

**Keywords:** collection, necrosectomy, pancreatitis, walled‐off necrosis

## Abstract

Pancreatitis is a condition much more commonly found in adults, but when diagnosed in the pediatric population, is often due to medications, congenital pathology, and critical illness. This patient had previously undergone treatment with 6‐mercaptopurine and presented with pancreatitis that eventually worsened to a walled‐off necrotic collection with paracolic extensions reaching the pelvis. Given clinical worsening with development of shock, procedural options for source control were weighed with gastroenterology, pediatric surgery, and interventional radiology, before pancreatic necrosectomy was determined to be the treatment of choice, given the adjacency of the collection to the stomach. A total of three separate endoscopic pancreatic necrosectomy procedures were performed and the patient s clinical status improved greatly, with vast improvement later seen on outpatient imaging. This successful treatment course argues for the efficacy of pancreatic necrosectomy even in very large walled off collections, and most importantly, lead to a positive outcome in this young patient.

## 1 BACKGROUND AND AIMS

Acute pancreatitis is a prominent pathology accounting for 275,000 hospitalizations a year in the United States.^1^ While pancreatitis in adult patients is most often caused by alcohol, pediatric etiologies by contrast are often related to medications, anatomic defects, and systemic infection.^2^, ^3^ In severe cases, large walled‐off necrosis can develop, potentially requiring drainage and/or necrosectomy for definitive treatment if there is mass effect or infection.^4^, ^5^, ^6^ We present a case of severe pancreatitis complicated by massive walled‐off necrosis and paracolic collection that was treated successfully with endoscopic necrosectomy.

## 2 CASE REPORT & PROCEDURE DESCRIPTION

**Figure 1:**
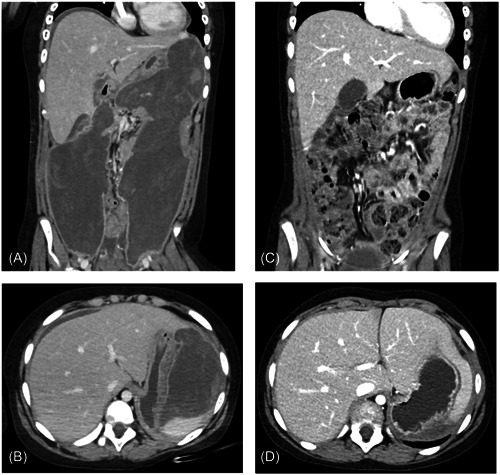
(A, B) Computed tomography (CT) abdomen and pelvis obtained upon admission, coronal and axial views, respectively. Evidence of severe pancreatitis with walled‐off necrosis in the bilateral paracolic gutters extending to the mid‐pelvis. (C,D) CT abdomen and pelvis obtained after discharge (53 days after the CT depicted in Images [A, B]) from the hospital. Significant interval improvement in both pancreatitis and in the size of the walled‐off collection is shown.

**Figure 2:**
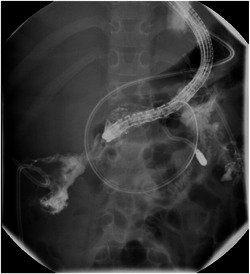
Injection of contrast on fluoroscopy revealing a continuous retroperitoneal space.

The patient is an 8‐year‐old female with a history of pre‐B‐cell acute lymphoblastic leukemia and recent severe pancreatitis secondary to 6‐mercaptopurine. She presented with systemic inflammatory response syndrome (SIRS), and notably a leukocytosis of 19.1 × 10^3^/µL, tachycardia, and temperature greater than 100.4 F. Computed tomography (CT) of the abdomen and pelvis revealed a multiloculated cavity in the retroperitoneum extending deep into the paracolic gutters (Video S1 and Figure 1A, B). Initial management was with naso‐jejunal feeds and antibiotics; however, due to clinical deterioration with increasing pressor requirements and persistent fevers, there was a concern for infected walled‐off collection and endoscopic drainage and necrosectomy were planned. Using a therapeutic endoscopic ultrasound (EUS) scope, one 20 mm × 10 mm Axios^TM^ stent was placed in the necrotic cavity and dilated to 20 mm. The endoscope was inserted through the Axios^TM^ and contrast was injected on the right and left, indicating continuity within the walled‐off collection (Video S1 and Figure 2). Initial mechanical and chemical necrosectomy was performed using endoscopic snare and hydrogen peroxide as can be seen in the video. The Axios^TM^ stent was left in place between each procedure and was not removed until after the final necrosectomy. Additionally, a pigtail catheter (converted from a colonic decompression catheter) was left in place traversing the Axios^TM^. Culture of the pancreatic collection fluid eventually grew *Candida glabrata* and the patient completed a course of piperacillin‐tazobactam empirically and fluconazole for fungal treatment, although blood cultures were negative for bacteremia and fungemia. The patient's symptoms continued to improve, and following discharge, interval CT abdomen and pelvis on Day 53 revealed vast improvement (Video S1 and Figure 1C, D). Outpatient follow‐up also revealed no evidence of pancreatic insufficiency, development of diabetes, or further abdominal pain.

## 3 DISCUSSION

This case demonstrates the effectiveness of endoscopic necrosectomy as the treatment for very large and severe cases of walled‐off necrosis (Figure 1A–D). While this patient was treated with empiric antibiotics and antifungals (given *C. glabrata* growth) as part of empiric treatment of shock, it is important to recognize that severe pancreatitis can present initially with SIRS and shock in the absence of infection since pancreatitis is a highly inflammatory state in itself.^2^ However, acute clinical worsening of status after the initial presentation should raise suspicion for infection. If a collection can be accessed from a luminal organ, EUS‐guided necrosectomy is often preferred and can avert the need for surgical or percutaneous drainage. Still, it is valuable and appropriate to discuss the approach for necrosectomy with colleagues from interventional radiology and surgery. From a technical standpoint, it is vital to have a well‐aligned initial puncture site through the stomach as this eases the passage of the endoscope. Contrast is also highly useful as this allows the endoscopist to better visualize the collection and “map” the cavity as can be seen in the accompanying video. It is important not to place arbitrary limitations on patients who may be candidates for endoscopic necrosectomy, even in very large collections with paracolic extension. Most importantly, this treatment led to a positive outcome in a pediatric patient and allowed her to avoid the risks of open surgery.

## CONFLICT OF INTEREST STATEMENT

The authors declare no conflict of interest.

## ETHICS STATEMENT

Patient consent for publication of case details was obtained for this case report. Informed consent from the patient and legal guardians was obtained for this report.

## Supporting information


**Supplementary Video:** Video description of the patient's presentation and treatment as well as follow‐up.
